# Efficacy and toxicity of neoadjuvant radiotherapy with concomitant dose escalation for rectal cancer

**DOI:** 10.3389/fonc.2025.1725405

**Published:** 2025-12-09

**Authors:** Peng Wang, Kai Liu, Xiaoliang Liu, Weiping Wang, Dingchao Liu, Yidi Wang, Shuai Sun, Ke Hu

**Affiliations:** Department of Radiation Oncology, Peking Union Medical College Hospital, Chinese Academy of Medical Sciences & Peking Union Medical College, Beijing, China

**Keywords:** rectal cancer, neoadjuvant chemoradiotherapy, concomitant dose escalation, pathological complete response, disease-free survival, toxicity

## Abstract

**Objective:**

This study aimed to evaluate the efficacy of a neoadjuvant radiotherapy regimen featuring a concomitant dose escalation regimen, followed by surgery, in patients with rectal cancer.

**Methods:**

We reviewed the clinical records of rectal cancer patients who received neoadjuvant chemoradiotherapy or radiotherapy alone with a concomitant dose escalation regimen (55–56 Gy) between October 2012 and January 2019. Chemotherapy regimens included capecitabine monotherapy or capecitabine combined with oxaliplatin. Surgery was performed 8–10 weeks after radiotherapy. Primary endpoints included the pathological complete response (pCR) rate and sphincter preservation rate. We also analyzed the impact of radiotherapy-to-surgery interval on pCR rates. Disease-free survival (DFS) was compared between pCR and non-pCR patients.

**Results:**

A total of 415 patients were included, with 14 patients received radiotherapy alone. Following neoadjuvant therapy with concomitant dose escalation and subsequent surgery, 99 patients achieved pCR, yielding a pCR rate of 23.9%. A complete primary tumor response (ypT0) was observed in 105 patients (a TpCR rate of 25.3%), while a nodal complete response (ypN0) was achieved in 335 patients (an NpCR rate of 80.2%). Among the 369 patients who underwent lymph node dissection, 295 achieved NpCR. Sphincter preservation was achieved in 359 (86.5%) patients The incidence of grade 3 or higher acute hematologic, gastrointestinal, and genitourinary toxicities was 9.7%, 4.2%, and 1.5%, respectively. With a median follow-up of 5.06 years, an interval between radiotherapy and surgery > 10 weeks was associated with a higher pCR rate (p=0.037) after adjusting for age and sex. Patients achieving pCR demonstrated significantly better DFS compared to non-pCR patients (83.33% vs. 62.55%, p=0.0224), and this difference remained significant in stage III patients (79.15% vs. 58.35%, p=0.0308).

**Conclusion:**

Neoadjuvant chemoradiotherapy with concomitant dose escalation yields high pCR rates for both primary tumor and nodal disease, with acceptable toxicity, in a significant number of patients with rectal cancer. Presence of pCR is associated with markedly improved DFS. Moreover, prolonged interval between neoadjuvant radiotherapy and surgery correlates with elevated pCR rates.

## Introduction

Neoadjuvant chemoradiotherapy followed by surgery is the current standard treatment for patients with locally advanced rectal cancer, as this approach has been shown to improve disease-free survival (DFS) and pathological complete response (pCR) rates without a significant increase in toxicity or surgical complications compared to postoperative chemoradiotherapy (1–4). In large-scale meta-analyses and clinical trials, standard neoadjuvant regimens typically resulted pCR rates ranging from 13% to 20%. For instance, a meta-analysis by Kong et al. involving 2,437 patients reported a pCR rate of 14.2%, while a large cohort study by Shin et al. and a retrospective study by Zhang et al. showed rates of 18.2% and 20.8%, respectively (5–7).

For radiotherapy, the most direct method to enhance efficacy is to increase the radiation dose. Concomitant dose escalation in rectal cancer treatment refers to increasing the radiation dose during neoadjuvant chemoradiotherapy, typically using advanced techniques such as intensity-modulated radiotherapy (IMRT), volumetric modulated arc therapy (VMAT), or image-guided radiotherapy (IGRT), with the goal of improving tumor response and local control while maintaining acceptable toxicity. The goal is to improve tumor response and local control while maintaining acceptable toxicity. However, recent clinical trials investigating this approach have produced divergent results. For example, the multicenter retrospective ATLANTIS part I study, which included 1,028 patients from 12 centers, demonstrated significantly higher pCR rates in patients receiving a dose boost compared to those who did not (26.6% vs. 17%, p<0.001) (8). In contrast, the Dutch RECTAL-BOOST phase II randomized controlled trial found no significant difference in clinical response (CR) rates after 50 Gy/25 fractions of external beam radiotherapy, regardless of whether an additional boost of 3 Gy×5 fractions was delivered (35.9% vs. 37.5%) (9). Further complicating the picture, the OPERA trial, a European multicenter phase III randomized trial, compared two boost methods in 141 patients: a 9 Gy external beam boost versus three 90 Gy high-dose-rate brachytherapy sessions. The results showed markedly different clinical complete response (cCR) or near-cCR rates (64% vs. 92%) and 3-year organ preservation rates (59% vs. 81%) between the two techniques (10).

Increasing evidence suggests that, compared to lower pCR rates with previous standard doses, preoperative neoadjuvant radiotherapy concomitant dose escalation (typically 54–60 Gy) using modern techniques can achieve pCR rates of approximately 20-28% in rectal cancer patients (11–15).Importantly, these higher response rates have been achieved without a significant increase in severe acute toxicity, with grade 3 or higher toxicity rates remaining around 10–11% (16).

Furthermore, higher pCR rates are associated with improved local control and may increase the likelihood of organ preservation (17). However, the impact of these improved pathological responses on long-term survival remains controversial. Studies have not consistently demonstrated that either concomitant dose escalation or the resultant higher pCR rates translate to improved overall survival. Indeed, some analyses report no significant differences in long-term survival between standard-dose and escalated-dose groups, despite the superior pCR rates in the latter (18).

Therefore, this study analyzed a cohort of rectal cancer patients treated with neoadjuvant chemoradiotherapy to evaluate the pCR rates, efficacy, and toxicity of a concomitant dose escalation protocol. The aim was to provide further clinical evidence to guide the use of this therapeutic strategy in the neoadjuvant setting for rectal cancer.

## Methods

### Study design and participant selection

This retrospective cohort study was approved by the Institutional Review Board of Peking Union Medical College Hospital, Chinese Academy of Medical Sciences, China (approval number S-K1330). We reviewed the clinical records of patients with rectal cancer who received neoadjuvant chemoradiotherapy or radiotherapy alone at our institution between October 2012 and January 2019. Inclusion criteria were as follows: (1) histologically confirmed rectal cancer, regardless of pathological types; (2) surgery performed 4 weeks – 15 months after completion of neoadjuvant treatment. Patients were excluded if they had missing data on digital rectal examination (DRE), complete blood count, biochemical analysis, tumor markers (CEA), colonoscopy, chest and abdomen computed tomography (CT) scan, rectum magnetic resonance imaging (MRI), transrectal ultrasound, and postoperative DFS. Positron emission tomography-CT (PET/CT) was not routinely performed.

### Radiotherapy

The clinical target volume (CTV) encompassed the entire mesorectum and pelvic lymph node regions (perirectal, obturator, presacral, internal iliac, partial external iliac, and common iliac), with modifications based on individual anatomy and disease extent. The gross tumor volume (GTV) included the primary tumor (GTV-T) and any positive pelvic lymph nodes (GTV-N), defined as nodes with a short-axis diameter ≥1 cm on MRI or PET/CT. A 7–10 mm margin was applied to the CTV to create the planning clinical target volume (PCTV), and a 5 mm margin was applied to the GTV to form the planning gross tumor volume (PGTV). These volumes were modified as needed to ensure that dose constraints for organs at risk were within acceptable limits.

The prescribed dose to the PCTV was 45–50 Gy in 25 fractions (5 fractions per week). A concomitant dose escalation was delivered to the primary tumor PGTV region, for a total dose of 55–56 Gy in 25 fractions. For both volumes, the treatment plan was designed to ensure that at least 95% of the volume received the prescribed dose. All eligible patients received IMRT with image guidance. Radiotherapy techniques included VMAT and Tomotherapy (TOMO). Image verification consisted of weekly cone-beam CT for VMAT patients and daily megavoltage CT for TOMO patients.

### Chemotherapy

Concurrent neoadjuvant chemotherapy regimens included oral capecitabine (825 mg/m^2^, day 1-14) with or without oxaliplatin (130 mg/m^2^, day 1, Xelox). Decisions regarding adjuvant chemotherapy were made postoperatively based on pathology and the patient’s clinical condition.

### Surgery

All patients underwent surgery 8–10 weeks after completing neoadjuvant treatment. Preoperatively, patients were reassessed with rectal CT, MRI, and/or transrectal ultrasound to evaluate tumor response and the feasibility of sphincter preservation. The surgical approach was determined based on the patient’s condition, personal preferences, and the attending surgeon’s judgment. The sphincter-preserving procedures included the Dixon procedure, intersphincteric resection with stoma, or transanal endoscopic microsurgery.

### Follow-up and toxicity evaluation

Follow-up schedule was scheduled as follows: every 3 months for the first 2 years, every 6 months for 3–5 years, and annually thereafter. Routine follow-up included DRE, complete blood count, liver and kidney function tests, CEA, colonoscopy, chest and abdominal CT, and pelvic MRI. Acute toxicities related to neoadjuvant treatment were graded using the Common Terminology Criteria for Adverse Events version 4.0.

### Statistical analyses

pCR is universally defined as no residual viable tumor cells in the surgical specimen, including both the primary rectal tumor site and all examined lymph nodes (ypT0N0) (19–21). DFS was defined as the time from the initiation of treatment to disease recurrence, death, or the date of the last follow-up. Patients were stratified into pCR and non-pCR groups based on postoperative pathology. All data were analyzed using R software (R version 4.3.1). Categorical variables (e.g., patient age, sex, stage) were compared using the Chi-squared test. Continuous variables (e.g., pretreatment CEA) were analyzed using the Wilcoxon rank-sum test to assess their impact on pCR. DFS was estimated using the Kaplan-Meier method, and survival curves were compared using the log-rank test. Cox proportional hazards models were used to compute hazard ratios (HR) and p-values. The association between the radiotherapy-to-surgery interval and pCR rates was also evaluated a multivariate logistic regression analysis. A p-value ≤0.05 was considered statistically significant.

## Results

### Patient characteristics

**Figure 1:**
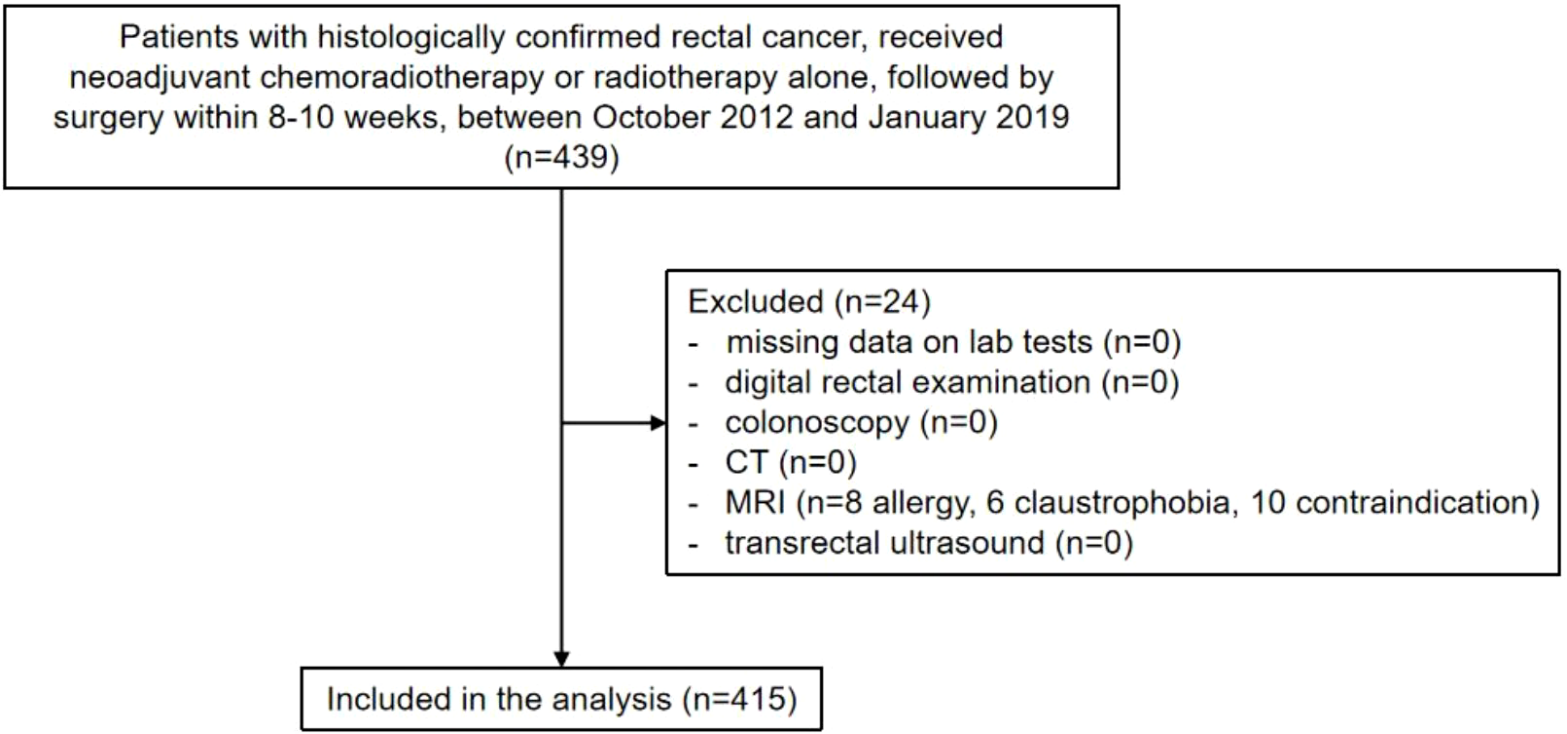
Participant selection flowchart. CT, computed tomography; MRI, magnetic resonance imaging.

A total of 415 patients with rectal cancer were included in this analysis (**Figure 1**). All patients received neoadjuvant chemoradiotherapy or radiotherapy with a concomitant dose escalation (55–56 Gy) to the primary tumor. There were 34.94% patients ≥ 65 years old, with 306 males (73.7%) and 109 females (26.3%). Tumor location distributions were 193 (46.5%) in the low rectum, 157 (37.8%) in the mid-rectum, and 14 (3.4%) in the upper rectum. Detailed patient characteristics are presented in **Table 1**.

**Table 1:** Patients characteristics.

Characteristics		No. (%)
Sex	Female	109 (26.27%)
	Male	306 (73.72%)
Age	< 65y	270 (65.06%)
	≥ 65y	145 (34.94%)
Tumor location	High	14 (3.37%)
	Medium	157 (37.83%)
	Low	193 (46.51%)
Stage	I	14 (3.37%)
	II	32 (7.71%)
	III	343 (82.65%)
	IV	6 (1.45%)
	T1	2 (0.48%)
	T2	31 (7.47%)
	T3	306 (73.73%)
	T4	67 (16.14%)
	Tx	9 (2.17%)
	N0	48 (11.5%)
	N1	145 (34.94%)
	N2	203 (48.92%)
	Nx	19 (4.58%)
Differentiation	High	55 (13.25%)
	Medium	227 (54.70%)
	Low	13 (3.13%)
CEA, ng/dL, mean ± standard deviation	16.34 ± 6.20	NA
Radiotherapy Techniques	IMRT	266 (64.10%)
	TOMO	148 (34.94%)
	Other	1 (0.24%)
Radiotherapy to surgery Interval	≤ 70d	334(80.49%)
	> 70d	81(19.51%)
Sphincter preservation	No	56 (13.49%)
	Yes	359 (86.51%)

IMRT, Intensity Modulated Radiation Therapy; TOMO, Tomotherapy.

In terms of pathological type of rectal cancer, 359 had adenocarcinoma, with remaining 56 having mucinous adenocarcinoma, squamous cell carcinoma, undifferentiated carcinoma, papillary carcinoma, adenosquamous carcinoma, signet-ring cell carcinoma, and unknown type.

### Neoadjuvant treatment

A total of 401 patients (96.6%) received concurrent chemotherapy with neoadjuvant radiotherapy, while the remaining 14 received radiotherapy alone due to patient-specific reasons. Among these 401 patients, 368 patients received 2–3 cycles of concurrent chemotherapy, while the remainder received individualized cycles administrated by their oncologists. Regarding radiation dose, all patients received a total dose of 55–56 Gy to the primary tumor region. Radiotherapy techniques included IMRT for 266 patients (64.1%), TOMO for 148 patients (35.7%), and other techniques for the remaining 14 patients. The median radiotherapy duration was 35 days, with 4 patients experiencing prolonged duration due to grade 3–4 hematological toxicity. Specific statistical results are shown in **Table 1**.

### Surgery

The median interval from the completion of radiotherapy to surgery was 9 weeks (range: 31–424 days). Sphincter preservation was achieved in 359 (86.5%) patients, while 56 (13.5%) patients underwent non-sphincter-preserving surgery based on surgeon evaluation and patient preferences. Further details are presented in **Table 1**.

### Treatment related toxicities and complications

Acute toxicity was defined as adverse events occurring during treatment or within 3 month post-treatment. Among 345 patients with available toxicity data, the overall incidence of grade 3 or higher toxicity was 8.1% in 9 patients. Specifically, the rates of grade 3 or higher acute hematologic, gastrointestinal, and genitourinary toxicities were 10.7%, 5.2%, and 1.8%, respectively. Postoperative complications occurred in 7 patients (1.7%) and included anastomotic leakage, intestinal obstruction, and intra-abdominal infection.

### Pathological outcomes

Following neoadjuvant therapy and surgery, pathological examination of all 415 patients revealed that 99 (23.9%) achieved pCR. A complete primary tumor response (ypT0) was observed in 105 patients (25.3%). Of the 370 patients who underwent lymph node dissection, 295 (79.7%) achieved a nodal pCR (ypN0). Detailed pathological outcomes are shown in **Table 2**, with results stratified by clinical stage presented in **Table 3**.

**Table 2:** Patient pathological complete response. .

Responses		No. (%)
pCR	No	316 (76.14%)
	Yes	99 (23.86%)
Primary tumor pCR	No	310 (74.70%)
	Yes	105 (25.30%)
Nodal pCR *	No	74 (17.83%)
	Yes	295 (71.08%)

pCR, pathological complete response.

*Only for patients received lymph node dissection.

**Table 3:** Pathological responses of patients with different stage of rectal cancer.

Responses	Stage	%
pCR	I	35.71%
	II	25.00%
	III	24.2%
	IV	16.67%
Primary tumor pCR	I	35.71%
	II	25.00%
	III	25.95%
	IV	16.67%
Nodal pCR	I	100%
	II	100%
	III	71.14.%
	IV	80.00%

pCR, pathological complete response.

*Only for patients received lymph node dissection.

When patients were stratified by the radiotherapy-to-surgery interval (≤10 weeks [n=334] vs. >10 weeks [n=81]), a longer interval was associated with a significantly higher pCR rate in the multivariate regression analysis after adjusting for age, sex, T stage, and N stage (p=0.043. **Table 4**). Additionally, patients who achieved pCR had significantly lower pre-treatment CEA levels compared to non-pCR patients (p=0.011).

**Table 4:** Multivariate regression analysis on association between radiotherapy-to-surgery interval and pCR.

Variables	Estimate	Standard error	*Z*	P
Constant	-15.8527	619.5767	-0.026	0.980
Interval*	0.5708	0.2819	2.024	0.043
Age	0.0143	0.0110	1.296	0.195
Sex	0.3185	0.2929	1.087	0.277
T stage†				
T2	14.0227	619.5762	0.023	0.982
T3	13.4005	619.5761	0.022	0.983
T4	12.9818	619.5762	0.021	0.983
N stage‡				
N1	0.0625	0.4075	0.153	0.878
N2	0.1234	0.4100	0.301	0.764

*, radiotherapy-to-surgery interval > 10 weeks, with ≤ 10 weeks as reference.

†, T0 as reference.

‡, N0 as reference.

### Follow-up survival analysis

The median follow-up was 5.96 years, with recurrence, metastasis, or death defining events; 56 patients (13.49%) experienced recurrence, and 76 (18.31%) experienced events, with distant metastases predominantly in the lungs and liver.

**Figure 2:**
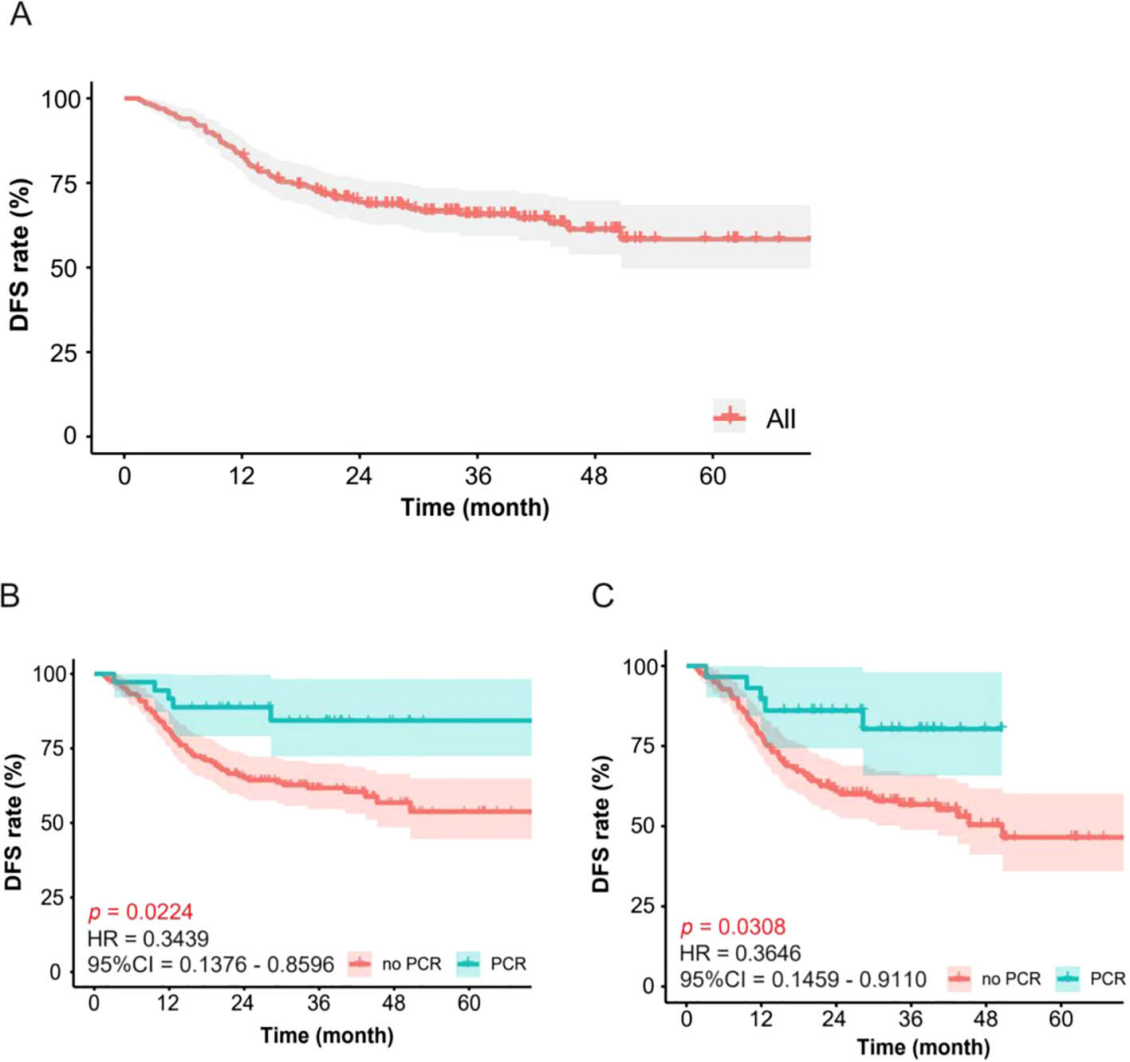
Disease-free survival (DFS) of patients with locally advanced rectal cancer treated with dose escalated radiotherapy followed by surgery. **(A)** DFS for all patients; **(B)** DFS in patients with pathological complete response (pCR)patients and non-pCR. The red line is the results of non-pCR patients. The green line is the results of pCR patients (p=0.0224); **(C)** DFS in patients of Stage III with pathological complete response (pCR) patients and non-pCR. The red line is the results of non-pCR patients.

Survival analysis yielded a 5-year DFS of 60.1% (see **Figure 2A**). Patients were then stratified by postoperative pCR status post-neoadjuvant treatment, revealing superior 3-year DFS in pCR versus non-pCR patients (83.33% vs. 62.55%, p=0.0224) (see **Figure 2B**). In this cohort, 343 patients (82.65%) were stage III; survival analysis in stage III pCR versus non-pCR groups showed significant 3-year DFS differences (79.15% vs. 58.35%, p=0.0308) (**Figure 2C**).

## Discussion

This study aimed to investigate whether a concomitant dose escalation to the primary tumor during neoadjuvant treatment could enhance therapeutic efficacy while maintaining a manageable safety profile. Our retrospective cohort analysis of 415 patients who completed this treatment regimen followed by surgery demonstrates several key findings. First, we demonstrated that pCR and downstaging of both T and N stages could be achieved in a significant number of patients undergoing concomitant dose escalation neoadjuvant therapy with subsequent surgery. Second, we found that a longer interval between the completion of radiotherapy and surgery was correlated with higher pCR rates. A crucial finding of this study is the strong association between achieving pCR and improved survival outcomes. Patients who achieved pCR had significantly better DFS compared to those who did not, a benefit that remained significant in the large subgroup of stage III patients. While the direct impact of concomitant dose escalation on survival requires further investigation, our results suggest it may confer a survival benefit. From a safety and quality-of-life perspective, the treatment was well-tolerated, and the high rate of sphincter preservation is a notable clinical benefit. Taken together, these findings support the scientific validity and feasibility of this approach, warranting further investigation.

Our study’s pCR rate of 23.86% is consistent with findings from other contemporary dose-escalation studies. For example, it is comparable to the multicenter ATLANTIS part I study, which reported a pCR rate of 26.6% in patients receiving a concomitant dose escalation, versus 17% in those who did not. The 17% pCR rate in the non-boosted arm of that study aligns with historical controls for standard neoadjuvant therapy (8). However, the literature is not uniformly supportive of this approach. Specifically, the Dutch RECTAL-BOOST study found no statistically significant difference in clinical response (CR) rates between groups with and without a dose boost after standard radiation (35.9% vs. 37.5%, respectively) (9). This discrepancy in the literature underscores that the impact of a concomitant dose escalation on pCR rates requires further investigation in prospective clinical trials.

A crucial question is whether the improved pCR rates from concomitant dose escalation translate into a direct survival benefit. While current prospective and retrospective cohort studies report that concomitant dose escalation can improve tumor downstaging, local control, and R0 resection rates, the dose-response relationship is not always linear, and higher doses (>58.9 Gy EQD2) may increase surgical complications, undermining patient long-term prognosis (13, 22). Furthermore, the data on survival are conflicting. Some retrospective and single-center studies suggest a positive impact on survival with concomitant dose escalation, but these findings are not universal and may be influenced by confounders such as patient selection or study design (23, 24). Conversely, large database analyses have even reported lower survival with increased doses, possibly due to such confounding factors (18). Therefore, while the ability of a concomitant dose escalation to improve pathological response is well-supported by evidence, its effect on long-term survival remains unconfirmed and inconsistent. Although modern techniques appear to have a manageable acute toxicity profile, the predominance of single-arm, heterogeneous studies limit definitive conclusions on survival. Consequently, it was suggested that additional studies are required to determine the long-term survival impact of concomitant dose escalation strategy (11). In the current study, we demonstrated a significant improved pCR in rectal cancer patients under concomitant dose escalation treatment, as well as a strong association between achieving pCR and superior DFS. The survival benefit was evident during postoperative 3-year follow-up period and in patients with stage III rectal cancer, providing a strong support that concomitant dose escalation strategy could be considered in rectal cancer patients.

Our study also contributes to the ongoing debate regarding the optimal interval between neoadjuvant radiotherapy and surgery. We found that an interval of more than 10 weeks was associated with a significantly higher pCR rate compared to an interval of 10 weeks or less (33.3% [27/81] vs. 21.6% [72/334]; p=0.037). This finding contrasts with some prior research, such as the 2016 GRECCAR-6 randomized controlled trial, which compared 7-week and 11-week intervals and found no significant difference in pCR rates (15.0% vs. 17.4%). Notably, that study reported that longer intervals were associated with higher morbidity and poorer surgical quality. Conversely, our results are supported by several meta-analyses indicating that extending the interval beyond the traditional 6–8 weeks after neoadjuvant chemoradiotherapy (CRT) significantly improves pCR rates (from approximately 13.7% to 19.5%) without increasing complications (25). Similarly, one systematic review found that waiting 8 weeks or more post-CRT leads to a significantly higher pCR rate (risk ratio 1.25) without increasing operative time or postoperative complications. However, this benefit does not appear to increase indefinitely with longer delay (26–28). Evidence suggests that pCR rates may peak at 10–12 weeks before stabilizing or declining, indicating that excessively long intervals may not provide additional advantages (29). All of above-mentioned studies did not use the concomitant dose escalation strategy during the neoadjuvant therapy for rectal cancer patients. In the current study, for the first time, we demonstrated that prolonging the interval between completion of neoadjuvant radiotherapy and surgery correlated with elevated pCR rates, implying that a longer preoperative interval might be recommended in these patients after concomitant dose escalation treatment.

In the future, the enhanced tumor response achieved with neoadjuvant concomitant dose escalation may play a crucial role in expanding non-operative management strategies for rectal cancer. The management of rectal cancer has evolved to include a “watch-and-wait” approach for patients who achieve a cCR after neoadjuvant chemoradiotherapy (CRT). Intensified regimens, such as those including a concomitant dose escalation or total neoadjuvant therapy (TNT), are increasingly used to maximize the likelihood of achieving cCR. Recent reviews and large studies indicate that carefully selected patients managed with this approach can have excellent 3-year overall survival rates of 92-94%. However, this strategy is associated with local recurrence rates of 15-22%, although most of these recurrences are salvageable with surgery (30, 31). While concomitant dose escalation and TNT regimens can further enhance cCR and organ preservation rates, they have not been shown to significantly affect disease-free or overall survival compared to standard management involving surgery (2, 5, 32, 33). The success of these organ-preserving strategies is highly dependent on stringent monitoring and careful patient selection, as patients with a poor or incomplete response have worse prognoses and should proceed to surgery without delay. Furthermore, long-term data and randomized trials specifically evaluating concomitant dose escalation as part of a non-surgical strategy remain limited, highlighting a critical need for ongoing research.

While concomitant dose escalation utilizing modern radiotherapy techniques is generally considered safe, rigorous patient selection is crucial. This is particularly important for elderly or frail patients, for whom higher doses may increase the risk of surgical complications and late toxicities (22, 34). Ongoing studies are focused on optimizing radiation dosing, techniques, and patient selection criteria to maximize therapeutic benefits while minimizing risks. The ultimate goal is to develop personalized treatment plans that enhance efficacy and improve patient quality of life. Although this study achieved higher pCR rates with concomitant dose escalation, its primary limitation is the lack of a control group. The retrospective study design and exclusion of patients with missing data could also introduce a potential selection bias into our analysis. In addition, we analyzed DFS but not overall survival in the current study. This could bring biases by limiting the ability to fully access the treatment long-term efficacy and safety. During the interval between neoadjuvant therapy and surgery, patients could receive additional cancer-related non-interventional treatment, which could certainly impact their prognosis. This was not analyzed in the current retrospective study due to limited available therapeutic data in the interval between neoadjuvant therapy and surgery. Therefore, future randomized controlled trials (RCTs) are needed for further validation.

## Conclusion

In conclusion, for patients with rectal cancer, neoadjuvant radiotherapy featuring a concomitant dose escalation is an effective strategy for achieving pCR with acceptable toxicities in a significant number of patients. Crucially, achieving pCR was associated with significantly improved DFS compared to a non-pCR outcome. Future RCTs are essential to definitively establish the role and long-term benefits of this concomitant dose escalation strategy.

## Data Availability

The raw data supporting the conclusions of this article will be made available by the authors, without undue reservation.
